# Antiretroviral Therapy Alters Skeletal Muscle DNA Methylation in HIV Mice and Promotes Cellular Senescence

**DOI:** 10.1093/geroni/igaf122.2672

**Published:** 2025-12-31

**Authors:** Alok Tripathi, Shabiha Sultana, Anthony Carrillo, Sadanand Fulzele, Huidong Shi, Eric Belin de Chantemele, Mark Hamrick, Meghan McGee Lawrence

**Affiliations:** Augusta University, Augusta, Georgia, United States; Augusta University, Augusta, Georgia, United States; Augusta University, Augusta, Georgia, United States; Augusta University, Augusta, Georgia, United States; Augusta University, Augusta, Georgia, United States; Augusta University, Augusta, Georgia, United States; Augusta University, Augusta, Georgia, United States; Augusta University, Augusta, Georgia, United States

## Abstract

HIV-associated morbidity and death have been decreased by antiretroviral therapy (ART), however age-related musculoskeletal frailty is exacerbated in people living with HIV (PLWH) and those on ART, contributing to poor healthspan. However, the combined effects of aging, HIV, and ART in musculoskeletal tissues have not been thoroughly studied. Here we investigated the effects of Emtricitabine (FTC), an FDA approved ART, or its vehicle (VEH) on skeletal muscle DNA methylation patterns in a female HIV transgenic mouse model (Tg26). Comparing the impact of HIV phenotypes in the absence of ART (i.e., WT+VEH vs. Tg26+VEH), 303 CpGs were significantly hypermethylated (methDiff≥0.1) whereas 273 were hypermethylated when comparing the same groups in the presence of ART (i.e., WT+FTC vs. Tg26+FTC). Mechanistically, the effects of FTC with TDF (Tenofovir, another ART) and TAF (Tenofovir alafenamide, a prodrug of TDF) as a combination ART (cART) were tested in primary human muscle cells. The cART treatment induced cellular senescence, (senescence-associated β-galactosidase staining) and qPCR suggested downregulated expression of genes involved in myoblast differentiation. These findings suggest that ART may have deleterious consequences on muscle function and may contribute to epigenetic changes in DNA methylation and cellular senescence, exacerbating muscle wasting and dysfunction in patients with HIV receiving ART treatments. Further research is needed to elucidate the mechanisms that drive muscle cell damage in PLWH on long-term cART and to understand contributions of altered DNA methylation to these processes. These insights could inform potential epigenetic-targeted therapeutic approaches for mitigation of musculoskeletal decline in PLWH on ART.

